# Anterior cervical discectomy and fusion with self-locking standalone cage for the treatment of cervical degenerative disc disease in patients over 80 years

**DOI:** 10.1186/s10195-025-00820-7

**Published:** 2025-01-29

**Authors:** Jian Zhou, An’nan Hu, Xiaogang Zhou, Jian Dong

**Affiliations:** Department of Orthopaedic Surgery, Zhongshan Hospital, Fudan University, 180 Fenglin Road, Shanghai, 200032 China

**Keywords:** Cervical degenerative disc disease, Anterior cervical discectomy, And fusion, Self-locking standalone cage, Over 80 years

## Abstract

**Background:**

The need for anterior cervical discectomy and fusion (ACDF) for cervical degenerative disc disease (CDDD) will probably grow dramatically in the geriatric population. However, ACDF with self-locking standalone cages in patients over 80 years has not yet been investigated. This study aimed to assess the clinical and radiographic results in patients over 80 years treated by ACDF with self-locking standalone cages.

**Methods:**

Between January 2018 and December 2019, patients with CDDD treated with ACDF were retrospectively stratified into two groups: the older group (≥ 80 years) and the younger group (< 65 years). The data collected included the demographics, preoperative comorbidities, intraoperative parameters, length of hospital stay, complications, clinical scores, and radiological parameters.

**Results:**

A total of 123 patients were included in the study. The mean follow-up duration was 28.3 ± 2.4 months. The hospital stay was 5.3 ± 0.6 days and 3.8 ± 0.4 days, respectively, for the older and younger groups. Postoperative complication rate was found higher in the older group than that of the young group without significance. All the patient-reported outcome parameters had significant improvement at the final follow-up. The two groups had no significant differences in terms of the excellent and reasonable rates, fusion rate, and the C2–C7 Cobb angle.

**Conclusions:**

Although a slightly higher incidence of complications, poorer recovery rate, and more extended hospital stay were found, without significant differences, satisfactory clinical and radiographic results were obtained in the older patients. The self-locking standalone cage is a safe and viable option for patients over 80 years who suffer from CDDD.

*Level of evidence* Level IV.

## Introduction

The number of older people has substantially increased over the last 10 years. It has been estimated that the worldwide population over 80 years of age will triple by 2050 [1]. Cervical degenerative disc disease (CDDD) is part of the natural aging process [2]. Surgery may be indicated if conservative treatment is unsuccessful or presents a crucial neurological deficit [3]. The surgical care for CDDD will probably grow for the next 3 decades because of this increase in the geriatric population and life span [4]. Anterior cervical discectomy and fusion (ACDF) were among the most commonly used surgical procedures for treating CDDD [5, 6]. It relieves neural compression and improves spinal stability by fusion of the affected segments and has demonstrated favorable outcomes [7]. However, previous studies found that advanced age is an independent risk factor for increased complications, greater morbidity, and longer hospitalizations than younger patients [8].

The cervical self-locking standalone cage (ROI-C, Zimmer Biomet, Austin, TX, USA) has been applied to treat the CDDD [9, 10]. Previous studies have shown the ROI-C cage allowed for similar or better clinical and radiographic outcomes compared with ACDF with anterior plating [11]. On the basis of randomized controlled trials, it revealed that the ROI-C cage is not only a safe and effective device for ACDF but also has the advantages of significantly reduced operation time, blood loss, overall incidence of dysphagia, and adjacent ligament ossification rate over anterior plating [12].

However, there has been a substantial lack of studies focused on older patients, specifically, those over 80 years who underwent the ACDF with a self-locking standalone cage. This study aimed to investigate postoperative clinical and radiographic results of ACDF with self-locking standalone cages in the patients and compare them with those of the younger patients.

## Materials and methods

The Institutional Ethics Committee of our hospital approved this retrospective and comparative clinical study.

### Patient population

Between January 2018 and December 2019, data from patients who suffered from CDDD and underwent ACDF with self-locking standalone cages at our institution were recruited. The inclusion criteria were set as follows: (1) patients with CDDD at levels from C3/C4 down to C6/C7 and patients presenting with radiculopathy and/or myelopathy with or without neck pain, (2) patients refractory to conservative treatment for at least 6 weeks, and (3) patients who received a minimum follow-up of more than 1 year. Patients were excluded if they: (1) had developmental stenosis and ossification of the posterior longitudinal ligament; (2) had had previous cervical operations and other cervical diseases, including trauma, tumor, and infection; and (3) required posterior surgery or simultaneous anterior and posterior surgery. On the basis of the aforementioned inclusion and exclusion criteria, 123 patients were included in the study. Patient symptoms included cervical radiculopathy and/or myelopathy. The radiologic diagnoses were established in each case via magnetic resonance imaging (MRI), three-dimensional computed tomography (CT), and radiographic studies of the cervical spine.

The study population was retrospectively stratified into two groups on the basis of whether the age at the time of surgery above 80 years (older group) or below 65 years (younger group).

### Surgical technique

All patients underwent ACDF performed by the senior surgeons in our hospital. The standard Smith–Robinson right-sided approach was performed. After identification and exposure of the relevant vertebral levels, adequate decompression of the spinal cord and nerve roots was ensured by excision of the herniated disc, osteophyte overgrowth on the uncovertebral joint and posterior lips of the vertebral body, and the posterior longitudinal ligament in all cases. After sufficient decompression, the cartilaginous endplate was removed entirely with the curette and high-speed burr to expose the cortical endplate. The cage size was determined by preoperative templating and intraoperative evaluation using a trial cage to confirm initial stability. Cages that fit tightly in the disc space without over-distraction of the disc space or facet joints were considered the correct height. The inner cavity of the cage was filled with excised local osteophytes and graft bone (Osteolink Biomaterial Co., Ltd., Hubei, China) in all patients. After implantation, two self-locking wings were placed into the upper and lower vertebra through the anterior part of the cage to ensure primary stabilization. Wound drainage was used in all the cases, and the drainage was removed 24–48 h after surgery. Postoperatively, all patients were encouraged to get off the bed after 24 h, and a soft collar was adopted for 6 weeks.

### Data collection and outcome evaluations

The data collected included the demographics, preoperative comorbidities (including diabetes, hypertension, pulmonary comorbidities, cardiac comorbidities, and cerebrovascular comorbidities), intraoperative parameters (intraoperative blood loss and operation time), length of hospital stay, complications, and clinical and radiological parameters.

The incidence of dysphagia was detected using the system defined by Bazaz [13]. The presence of dysphagia was evaluated at 1 week postoperatively and at 3 months postoperatively. In addition, any complications occurring during the hospitalization were recorded.

The clinical scores and radiographic parameters were collected before surgery, 1 week after operation, at 3 and 6 months post-operation, and every year after that.

Before surgery and each follow-up, the clinical scores were acquired by asking patients to complete the questionnaires, including visual analogue scale (VAS) score questionnaires and a Japanese Orthopedic Association (JOA) score questionnaire. The JOA recovery rate was calculated using the formula: (postoperative score − preoperative score) 100/[17 (total score) − preoperative score]. Odom’s grading system was used to evaluate patient satisfaction with the surgery.

Radiological evaluation included anterior–posterior and lateral X-rays, with flexion–extension views, CT, and MRI. Cervical lordosis was assessed using the C2–C7 Cobb angle. Two-dimensional CT reconstruction was performed when evidence of bone fusion could be observed in the X-ray examination. Fusion in each level was assessed using all the following criteria: (1) the absence of motion > 2 mm between the spinous processes on flexion–extension lateral radiographs, (2) the absence of a radiolucent gap between the graft and the endplate, and (3) the presence of continuous bridging trabeculae at the graft and endplate junction. The radiographic subsidence was defined as cage migration of 3 mm or more into the adjacent vertebral body. To correct for intra-observer and inter-observer differences in radiological measurements, three experienced observers independently evaluated radiological outcomes.

### Statistical analysis

A standard SPSS software package (SPSS, Chicago, Illinois, USA) was used for the statistical analysis. Continuous variables are presented as means ± standard deviations and categorical variables as percentages. The chi-squared test was used to compare categorical variables between groups. Independent sample *t*-tests were performed to compare continuous data between the groups, and a paired *t*-test was used to evaluate differences from preoperative to postoperative time points. A *P*-value of less than 0.05 was considered statistically significant.

## Results

**Fig. 1:**
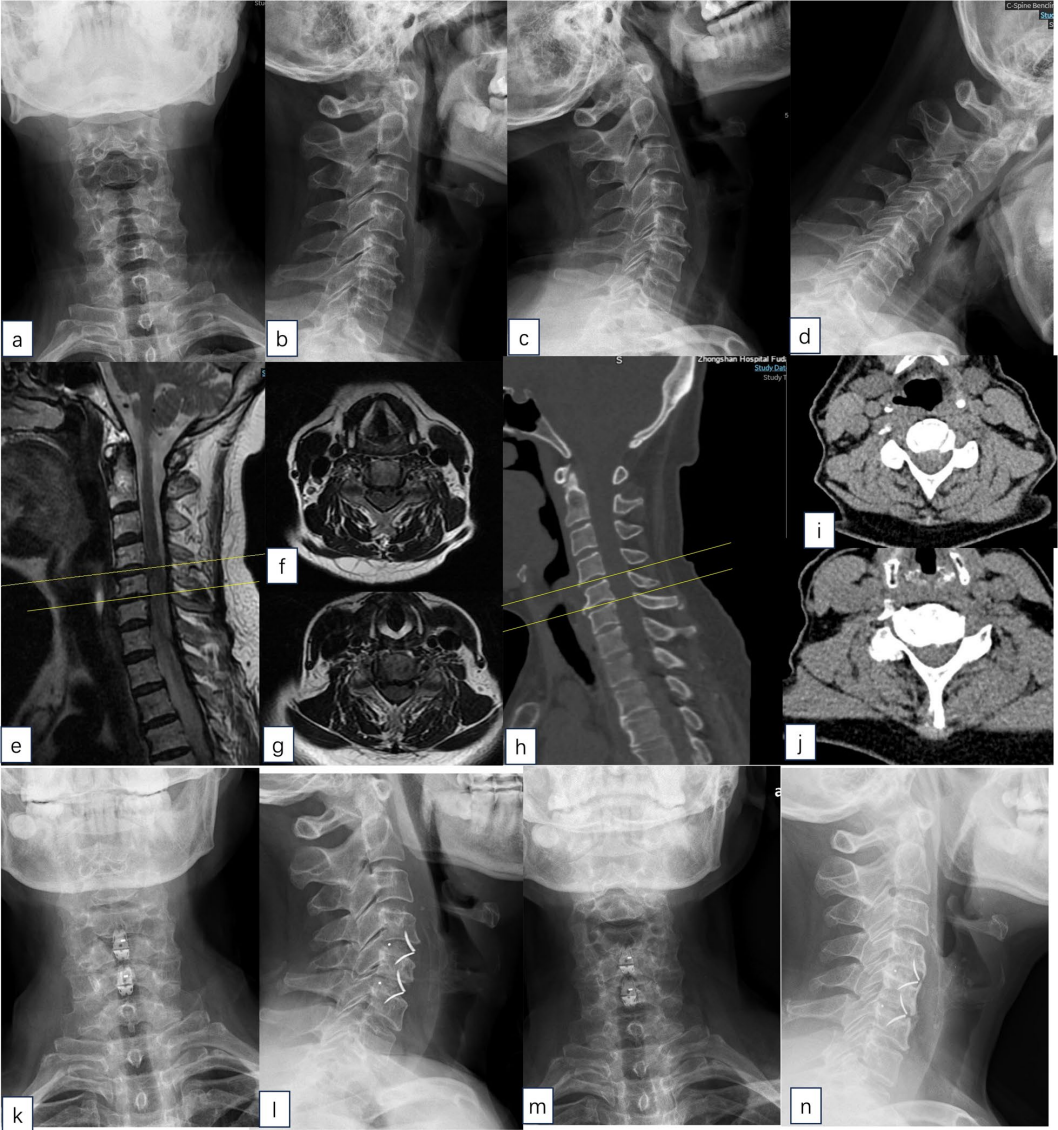
A patient aged 83 years old, who had symptoms of cervical radiculopathy and myelopathy, was treated with two-level ACDF with self-locking standalone cages. **a** and **b** Preoperative anteroposterior and lateral X-rays. **c** and **d** Flexion–extension cervical spine radiography. **e**–**g** Preoperative MRI showed C45 and C56 spinal stenosis and disc herniation. **h**–**j** Preoperative CT showed C45 and C56 spinal stenosis and soft disc herniation. **k** and **i** Anteroposterior and lateral X-rays at 3 days after the operation. **m** and **n** Anteroposterior and lateral X-rays at the final follow-up

The surgeries were successfully performed in all cases. An illustrative case was shown in the Fig. 1. The mean follow-up duration was 28.3 ± 2.4 months, without differences between the two groups (average of 22.6 versus 30.8 months in the older versus younger groups, respectively). None of the patients or data were lost during the follow-up. No patient underwent repeated surgery.

### Demographics, preoperative comorbidities, and intraoperative parameters

The demographic data are demonstrated in Table 1. The older group consisted of 51 patients (30 males and 21 females), with a mean age of 83.2 ± 2.53 years. The younger group comprised 72 patients (39 males and 33 females), with a mean age of 57.4 ± 3.7 years old. In the older group, 39 patients had chronic diseases. In all, 12 patients had one chronic disease, 21 had two chronic diseases, and 6 had three or more chronic diseases. In the young group, 24 patients had chronic diseases. The prevalence of chronic diseases in the older group was significantly greater than in the younger group. The demographic and clinical data were comparable in the two groups. The most commonly indexed fusion levels were C4–5 and C5–6, accounting for 67.9% of the entire series. However, there were no differences in the level of distribution, intraoperative blood loss, and operative time (Table 2).

**Table 1:** The demographic data, clinical data, and preoperative comorbidities in both groups

			Younger group (< 65 years)	Older group (≥ 80 years)	*P*-value
Sex	Male		39 (54.17%)	30 (58.82%)	0.065
	Female		33 (45.83%)	21 (41.18%)	0.059
Age			57.4 ± 3.7	83.2 ± 2.53	**0.032**
Operated level	C34		19 (14.96%)	13 (13.83%)	0.068
	C45		49 (38.58%)	36 (38.30%)	0.076
	C56		38 (29.92%)	27 (28.72%)	0.064
	C67		21 (16.54%)	18 (19.15%)	0.059
Number of treated levels	One level		29 (40.28%)	18 (35.29%)	0.061
	Two levels		31 (43.06%)	23 (45.10%)	0.069
	Three levels		12 (16.66%)	10 (19.61%)	0.062
Preoperative comorbidities	Patients		24 (33.33%)	39 (76.47%)	**0.036**
	Disease	Diabetes	12	23	
		Hypertension	7	18	
		Pulmonary comorbidity	4	11	
		Cardiac comorbidity	5	13	
		Cerebrovascular comorbidity	2	9	
Symptoms	Myleopathy		24 (33.33%)	19 (37.25%)	0.071
	Radiculopathy		38 (52.78%)	26 (50.98%)	0.065
	Myleopathy and radiculopathy		10 (13.89%)	6 (11.76%)	0.063

**Table 2:** Intraoperative parameters, hospital stay, and complications in both groups

		Younger group (< 65 years)	Older group (≥ 80 years)	*P*-value
Operation time		75 ± 12.5 min	69 ± 10.9 min	0.075
Intraoperative blood loss		85 ± 11 ml	76 ± 9 ml	0.083
Hospital stay		3.8 ± 0.4 days	5.3 ± 0.6 days	0.056
Perioperative complications		10 (13.89%)	10 (19.61%)	0.054
Dura tear		3	1	
Dysphagia		3	3	
Pneumonia		1	1	
Deep vein thrombosis		1	1	
Urinary tract infection		2	3	
Cerebral infarction		0	1	
Fusion rate		93.1%	88.2%	0.7155
Fusion time		9.0 ± 3.6 months	9.7 ± 3.9 months	0.248

### Hospital stay

The patients in the older group were hospitalized for 4–6 days, with a mean of 5.3 ± 0.6 days. The hospital stay of the younger group was 3–5 days, with a mean of 3.8 ± 0.4 days.

### Complications

After surgery, none of the patients suffered from neurological deterioration. There were no infections or epidural hematoma in either group. Moreover, the two groups had no implant dislodgement, malposition, or hardware breakage during the follow-up period.

The complication profile is displayed in Table 2. Postoperative complications were found in 19.61% of patients in the older group and 13.89% in the younger group. Postoperative complications were slightly more common in the older group than in the younger group without significant differences (*P* = 0.054). Urinary tract infection and dysphagia were the most common postoperative complications that occurred in the older group.

There was a slightly higher incidence of dysphagia in the older group, but it reached no statistical significance compared with the young group (*P* = 0.064). Transient postoperative dysphagia (< 3 months) was found in 4.17% of patients in the younger group and 5.88% of patients in the older group. Although complaints of dysphagia persisted beyond 3 months in only one patient in the younger group, they were recorded in two patients in the older group.

### Clinical and radiological outcomes

The clinical outcomes of these patients, including VAS and JOA scores, are summarized in Table 3. All the patient-reported outcome parameters significantly improved at the final follow-up compared to the pre-operative scores. There were no significant differences between the two groups in the VAS and JOA scores at any time point (all *P* > 0.05). No significant difference was between the older and younger groups regarding the JOA recovery rate. Based on Odom’s criteria, none of the patients in both groups experienced a poor clinical outcome. There were no significant differences between the two groups’ excellent and reasonable rates.

**Table 3:** Clinical results in both groups

	Younger group (< 65 years)	Older group (> 80 years)	*P*-value
VAS			
Preoperative	7.3 ± 0.4	7.6 ± 0.3	0.068
Postoperative	2.1 ± 0.3	2.5 ± 0.2	0.064
3 months after operation	1.9 ± 0.1	2.1 ± 0.2	0.071
Final follow-up	1.2 ± 0.2	1.1 ± 0.3	0.076
JOA			
Preoperative	10.3 ± 2.1	10.7 ± 1.9	0.066
Postoperative	15.2 ± 2.3	14.7 ± 2.2	0.067
3 months after operation	16.1 ± 1.3	15.4 ± 1.5	0.059
Final follow-up	16.2 ± 1.9	15.6 ± 1.7	0.071
JOA recovery rate	73.1 ± 2.1%	68.5 ± 1.9%	0.053
Odom’s grading			
Excellent	50 (69.44%)	30 (58.82%)	0.052
Good	14 (19.45%)	11 (21.57%)	0.054
Fair	8 (11.11%)	10 (19.61%)	0.048
Poor	0	0	

**Fig. 2:**
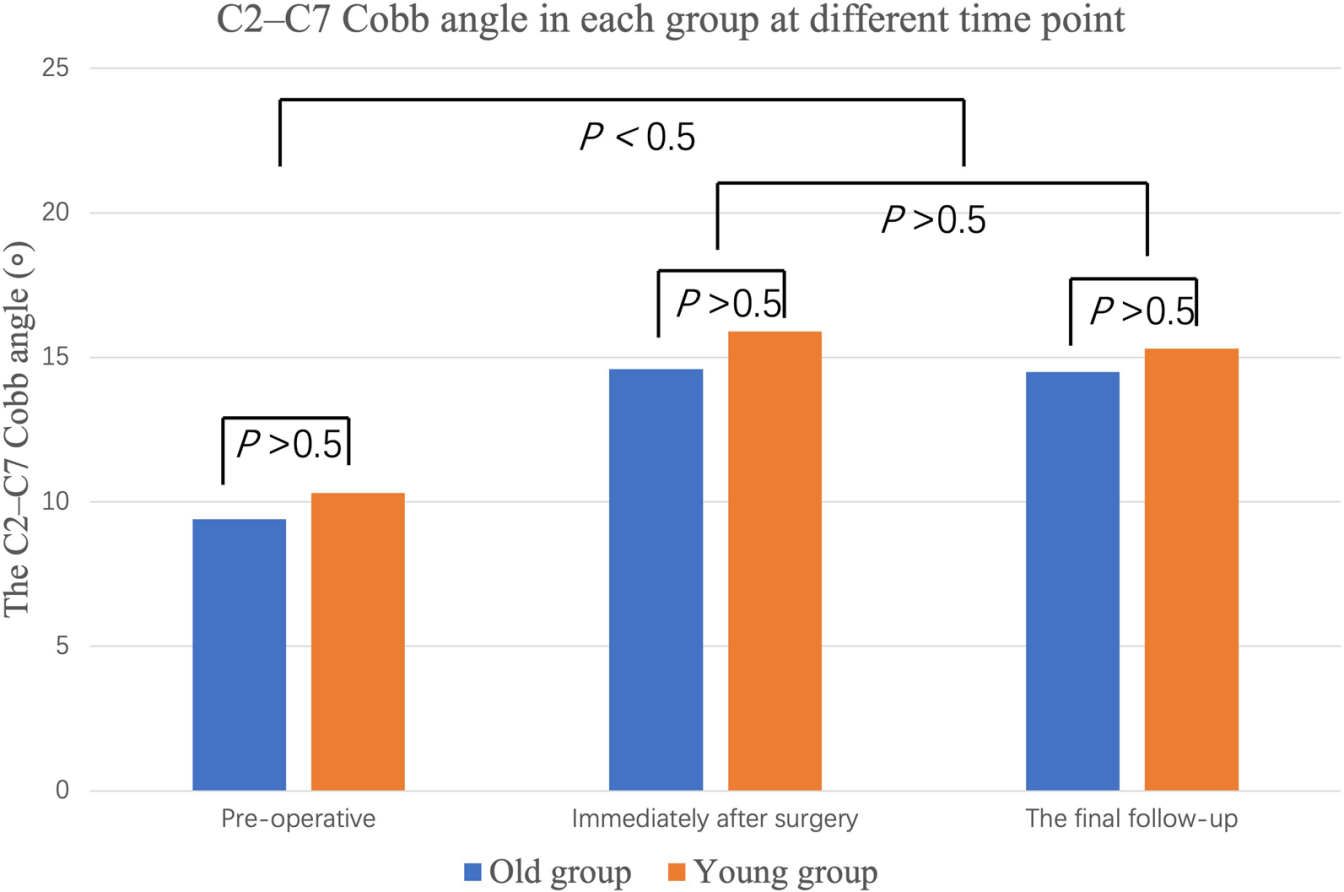
The C2–C7 Cobb angle of all patients in both groups improved significantly after surgery at any time point compared with the preoperative ones (*P* < 0.05). There were no significant differences between the two groups in the C2–C7 Cobb angle at any time point (all *P* > 0.05)

The C2–C7 Cobb angle of all patients in both groups improved significantly after surgery at any time point compared with the preoperative ones (all *P* < 0.05; Fig. 2). The C2–C7 Cobb angle showed no significant difference between postoperative time points and the last follow-up in each group (all *P* > 0.05). There were no significant differences between the two groups in the C2–C7 Cobb angle at any time point (all *P* > 0.05).

At final follow-up, the fusion rates were 88.2% in the older group and 93.1% in the younger group, which showed no significant difference between groups (*P* = 0.7155). The radiologic mean fusion time was 9.7 ± 3.9 months in the older group and 9.0 ± 3.6 months in the younger group. Although the radiologic mean fusion time was slightly prolonged in the older group compared with the younger group, no significant difference (*P* = 0.248) was noted.

In the older group, six cages in four patients subsided. In the younger group, two cages in two patients subsided. All these patients with cage subsidence did not experience any symptoms, and they achieved fusion in the final follow-up.

## Discussion

The self-locking standalone cage is introduced and applied in the ACDF to reduce complications, such as soft-tissue damage, chronic dysphagia, and adjacent segment degeneration, that happen in anterior plate fixation. It is widely accepted that adopting the self-locking standalone cage can decrease blood loss, operative time, and length of hospital stay; result in higher JOA scores; effectively restore the cervical physiological curvature; and lead to satisfactory outcomes [14–16].

Because there is a trend of population aging and prolongation of healthy life expectancy, the chance of the elderly undergoing cervical surgery is expected to increase. Generally, older age is a factor in blood transfusions, reoperations, extended length of stays, and increased postoperative complications. Consequently, comprehensive work is necessary to analyze the efficacy the self-locking standalone cage in older patients undergoing ACDF procedures. However, in the previous studies of the self-locking standalone cage, patients’ ages were almost all under 80 years [16–19]. With the life span increasing, an increasing number of older patients receive ACDF, which gives us a chance to investigate the outcome and complications of ACDF with the self-locking standalone cage in patients over 80 years.

The present study showed no significant differences in the radiological outcomes between the older and younger groups. In all, 89.2% of patients in the older group and 92.9% in the younger group got solid fusion at the final follow-up. The mean time to achieve a solid fusion was longer in the older group without significant differences. Six cages in four patients subsided in the older group, and two cages in two patients subsided in the younger group. These slight differences in the radiological outcomes may be due to the osteoporosis in the older group. It has been demonstrated that patients with osteoporosis experience slower and less reliable bone healing, increased risk of interbody cage subsidence, pseudoarthrosis, and progressive kyphosis. Using a self-locking standalone cage is also a factor of subsidence compared with the plate-cage system [9]. However, only four older and two younger patients had subsidence in the current study. Several optimal surgical techniques were adopted in our research. First, the bony endplate was preserved as much as possible. Second, the cage size should be carefully tested before installation, and segmental overdistraction should be avoided. Furthermore, the modulus of the elasticity of polyetheretherketone (PEEK) is similar to that of bone. Consequently, in our study, a small percentage of cages subsided; all the involved patients reported good clinical outcomes.

The neurological functions and symptoms of all the patients in both groups were relieved according to the lower VAS and higher JOA scores evaluated after the operation. The self-locking standalone cage decreases blood loss and operative time. It makes it possible for surgeons to thoroughly remove the compressive tissue and decompress the affected nerve, even in older patients. Although the postoperative VAS and JOA evaluations of the older group are slightly worse than those of the younger group, no significant difference can be found between the two groups.

In some studies, complications and readmissions are more likely to happen in older patients [20, 21]. However, a previous study showed prolonged operative time was associated with an increased risk of complications in the elderly population [22]. It has been demonstrated that the self-locking standalone cage obviously decreases operative duration in patients with traditional anterior cervical plating systems [10, 23]. In this study, the operative duration was comparable in the two groups. Consequently, 19.61% of older and 13.89% of younger patients suffered complications without significant differences in the current study.

Dysphagia is a common complication in ACDF patients, with an incidence between 1 and 79% [24]. Patients undergoing ACDF with anterior plate fixation are more likely to have dysphagia compared with those without plate fixation [25]. Wang et al. believed a zero-profile anchored spacer was associated with a lower risk of postoperative dysphagia for less foreign body stimulation [26]. However, Chen et al. indicated that less traction time and less damage to prevertebral soft tissues during surgery contributed to the relatively low rate of dysphagia [9]. Singh et al. demonstrated that age is an independent predictor for dysphagia in ACDF patients [27]. In pathophysiology, frailty and muscular, endocrine, or psychiatric diseases may contribute to dysphagia in the older population [28]. In this study, a slightly higher dysphagia rate was found in the older group, implying that patients over 80 years still have a higher incidence of dysphagia regardless, which is greatly lessened by using a standalone self-lock cage.

This study is not without limitations. First, the retrospective nature of this study may place the analysis at risk of selection bias. Second, the sample size was small. Third, all patients were evaluated and operated in a single center, thus limiting the generalizability of the study findings. Therefore, large-scale, prospective studies with more details on comorbidities and complications are critical to overcome these limitations.

## Conclusions

Although a slightly higher incidence of complications, poorer recovery rate, and more extended hospital stay were found, without significant differences, the satisfactory clinical and radiographic results were obtained in the older patients. The self-locking standalone cage is a safe and viable option for patients over 80 years who suffer from CDDD.

## Data Availability

Not applicable.

## Abbreviations

CDDD: Cervical degenerative disc disease
ACDF: Anterior cervical discectomy and fusion
MRI: Magnetic resonance imaging
CT: Computed tomography
VAS: Visual analogue scale
JOA: Japanese Orthopedic Association
